# Anatomical, pathological, and histological features of experimental respiratory infection of birds by biofilm-forming bacteria *Staphylococcus aureus*

**DOI:** 10.14202/vetworld.2024.612-619

**Published:** 2024-03-17

**Authors:** Ekaterina Lenchenko, Nadezhda Sachivkina, Olesya Petrukhina, Nikolay Petukhov, Andrey Zharov, Natallia Zhabo, Marina Avdonina

**Affiliations:** 1 Department of Veterinary Medicine, Russian Biotechnological University (BIOTECH University), 125080, Moscow, Russia; 2 Department of Veterinary Medicine, Agrarian Technological Institute, Peoples’ Friendship University of Russia (RUDN University), 117198 Moscow, Russia; 3 Department of Technosphere Security, Agrarian Technological Institute, Peoples’ Friendship University of Russia (RUDN University), 117198, Moscow, Russia; 4 Department of Foreign Languages, Institute of Medicine, Peoples’ Friendship University of Russia (RUDN University), 117198, Moscow, Russia; 5 Department of Linguistics and Intercultural Communication of the Faculty of Distance Learning and Part-Time Education of Moscow State Linguistic University, 119034 Moscow, Russia

**Keywords:** airsacculitis, avian, biofilm, pathogenesis, respiratory syndrome pneumonia, *Staphylococcus aureus*

## Abstract

**Background and Aim:**

The pathogenesis of staphylococcal infections is mediated by virulence factors, such as enzymes, toxins, and biofilms, which increase the resistance of microorganisms to host immune system evasion. Testing and searching for standardized multi-level algorithms for the indication and differentiation of biofilms at the early stages of diagnosis will contribute to the development of preventive measures to control the critical points of technology and manage dangerous risk factors for the spread of infectious diseases. This research aimed to study the main stages of *Staphylococcus aureu*s biofilm formation in *in vitro* experiments and to analyze the dynamics of respiratory syndrome development in chickens infected with these bacteria.

**Materials and Methods:**

Experimental reproduction of the infectious process was performed using laboratory models: 10-day-old White Leghorn chickens (n = 20). Before the experiments, the birds were divided into two groups according to the principle of analogs: Group I (control, n = 10): the birds were intranasally inoculated with 0.5 cm^3^ of 0.9% NaCl solution; Group II (experiment, n = 10): the birds were intranasally inoculated with a suspension of *S. aureus* bacteria, 0.5 cm^3^, concentration 1 billion/cm^3^.

**Results:**

Colonization of individual areas of the substrate under study *in vitro* occurred gradually from the sedimentation and adhesion of single motile planktonic cells to the attachment stage of microcolony development. Staining preparations with gentian violet due to the “metachromosia” property of this dye are a quick and fairly simple way to differentiate cells and the intercellular matrix of biofilms. Fixation with vapors of glutaraldehyde and osmium tetroxide preserves the natural architecture of biofilms under optical and scanning electron microscopy. Pure cultures of *S. aureus* microorganisms were isolated from the blood, lungs, small intestine, liver, kidneys, and spleen after 5–10 days during experimental infection of chickens. Clinical signs of respiratory syndrome developed within 5–6 days after infection. Acute and subacute serous-fibrinous airsacculitis, characterized by edema and thickening of the membranes of the air sacs and the presence of turbid, watery, foamy contents in the cavity, was the most characteristic pathomorphological sign. The signs of acute congestive hyperemia and one-sided serous-fibrinous pneumonia developed with significant thickening of fibrinous deposits. In Garder’s gland, there was an increase in the number of secretory sections, indicating hypersecretion of the glands. In the lymphoid follicles of Meckel’s diverticulum, leukocytes, usually lymphocytes, and pseudoeosinophils were detected.

**Conclusions:**

Hydration and heteromorphism of the internal environment of biofilms determine the localization of differentiated cells in a three-dimensional matrix for protection against adverse factors. The most characteristic pathomorphological sign was the development of acute and subacute serous-fibrinous airsacculitis when reproducing the infectious process in susceptible models. There was a significant thickening of fibrinous deposits and signs of acute congestive hyperemia and one or two serous-fibrinous pneumonia developed.

## Introduction

Throughout the world, there is an increasing trend in the statistically significant registration of nosocomial pneumonia, endocarditis, and sepsis mediated by the formation of *Staphylococcus aureus* biofilm [1]. These bacteria may cause pyoderma, endocarditis, osteomyelitis, sepsis, pneumonia, toxic shock syndrome, and food poisoning [2, 3]. *S. aureus* is associated with pneumonia, sepsis, septic arthritis, and brain abscess [4]. The incidence of methicillin-resistant *S. aureus* (MRSA)-associated surgical infections is associated with long-term catheter use [5]. The pathogenesis of staphylococcal infections is mediated by virulence factors, such as enzymes, toxins, and biofilms, which increase the resistance of microorganisms that evade the host immune system [6].

*S. aureus* is a cause of animal diseases, particularly significant economic losses for the global broiler chicken industry [7]. *S. aureus* strains isolated from pathological avian material and environmental objects of poultry farms and poultry processing enterprises exhibit a high frequency of biofilm formation [8]. *Staphylococcus* spp. microorganisms, mainly *S. aureus* (38.9%), have been identified in tenosynovitis, arthritis, and osteomyelitis of the femur or tibia of broilers aged 9–21 weeks [9]. Bacterial isolates from the ovarian follicles of sick chickens were identified mainly in the form of associations; virulent *S. aureus* and hemolytic *Escherichia coli* strains were isolated to a greater extent [10]. Exogenous staphylococcal avian infections develop when skin, mucous membrane, and vitelline duct of chickens are damaged; endogenous infections occur during colonization of the respiratory tract and subsequent septicemia [11]. Systemic staphylococcal infection, manifested by swelling of the combs, fever, and decreased egg production after molting of laying hens, is facilitated by age-related immune suppression [12]. Focal necrotizing dermatitis at the base of the ridge of birds is characterized by the formation of a typical granuloma with visible bacterial colonies of large cocci [13]. *S. aureus* was isolated from the pathological material of psittacine birds with lesions characteristic of septicemia, and coagulase-negative staphylococci, streptococci, *Chlamydophila*, *Enterococcus hirae*, and *Candida* were also identified [14]. MRSA was isolated from a swab of a non-healing wound on the lateral thigh of a 4-year-old male canary, *Serinus canaria* domestica [15]. The following phenotypic/percentage/gene indicators were detected in *S. aureus* from nasal and tracheal washings of Ciconia storks: penicillin/79.1%/*blaZ*; erythromycin-clindamycin-inducible/19.1%/*ermA*; *ermT*; tetracycline/11.9%/*tetK*; clindamycin/4.5%/*lnuA*; and ciprofloxacin/4.5% [16].

Massive respiratory infections of bacterial, viral, and mycological etiology develop as a result of violations of bird-keeping technology, non-compliance with air exchange parameters, temperature and humidity conditions, increased concentrations of harmful gases, and unbalanced nutrition [17–20]. Chickens aged 1–2 months and laying hens are most susceptible to respiratory infections [21]. Broiler chickens with signs of airsacculitis had a lower body weight and higher fecal contamination than chickens without signs of airsacculitis. Accordingly, the risk of bacterial contamination of carcasses increases, which is a factor in the transmission of pathogens of foodborne toxic infections to humans [22].

Due to the increasing resistance of *S. aureus* to various antibiotics used in the poultry industry, great efforts have been made to identify new compounds or therapeutic strategies that can replace or reduce the number of antibacterial drugs used. In particular, researchers are interested in strategies to reduce biofilm formation and effectively treat biofilms once they form [23]. Adhesion is a stage of sedimentation and the primary, so-called reversible attachment of planktonic organisms. The reversible adhesion of planktonic forms of bacteria is gradually replaced by deformation of the cell wall and attachment of bacteria to the surface. The irreversible adhesion of microorganism fixation is observed during the secretion of exocellular polymers, mainly polysaccharides. Therefore, most researchers argue that it is best to fight biofilm-forming bacteria in the first stages of attachment to animal body cells [24, 25]. Staining smears with gentian violet is a very effective and inexpensive method due to the properties of this dye: “metachromosia” is a fast and fairly simple way to differentiate cells and the intercellular matrix of biofilms [26].

This research aimed to study the main stages of *S. aureus* biofilm formation in *in* *vitro* experiments and to analyze the dynamics of respiratory syndrome development in chickens infected with these bacteria. Applied aspects of studying the main stages of biofilm formation and experimental reproduction of the infectious process on susceptible models will contribute to the development of rational chemotherapeutic and disinfectant drug dosing regimens. On the basis of our experiment and analysis of similar data taken from the world scientific literature, the development patterns of pathogenic microorganism populations *in vitro* and *in vivo* are discussed.

## Materials and Methods

### Ethical approval

This study complied with the requirements of the “Directive 2010/63/EU of the European Parliament and the Council of the European Union” (September 22, 2010) on the protection of animals used for scientific purposes. All bird experiments followed the Guide for the care and use of laboratory animals (Committee for the Update) [27]. The birds were manipulated according to the Local Ethics Committee for animal experimentation, Peoples’ Friendship University of Russia (protocol number 114a; September 10, 2022).

### Study period and location

The study was conducted from September 01, 2022, to September 20, 2022, at the experimental base of Russian Biotechnological University (BIOTECH) and the Peoples’ Friendship University of Russia.

### Bacterial strains and media

The reference strain *S. aureus* American type culture collection (ATCC) 25923, obtained from the collection of L.A. Tarasevich State Research Institute for Standardization and Control of Medical Biological Preparations (Moscow, Russia) was used in this study [28]. Microorganism cultures were freeze-dried in 0.5% semi-liquid meat-peptone agar at 4°C ± 1°C. Microorganisms were cultured at 37 ± 1°C for 24 or 48 h on Blood Agar (Biomerieux, Marcy l’Etoile, France), Nutrient Broth and Baird–Parker Agar (HiMedia™ Laboratories Pvt. Ltd., Mumbai, India)) with 50 mL egg yolk tellurite emulsion (Merck, Darmstadt, Germany). Identification of *S. aureus* was confirmed using API (analytical profile index) Staph (Biomerieux).

### Phenotypic characterization of bacteria

The morphological, cultural, and chemical properties of microorganisms have been studied by generally accepted methods using differential diagnostic media and test systems [29, 30]. Bacterial phenotypes of the strains used in the experiment corresponded to Bergey’s classification from 1984 to 1989 [31].

### Morphometric and densitometric indicators of the bacterial biofilms

Morphometric indicators of biofilms were studied using 12-well plates (6.8 mL, Medpolymer, Moscow, Russia). Glasses measuring 18.0 × 18.0 mm were placed at the bottom of the wells of the plates for microscopic examination (Corning Inc., New York, USA). Later, 3.0 mL of nutrient broth and 1.0 mL of a bacterial suspension at a concentration of 0.5 units (McFarland) were placed in the wells. Microorganisms were cultured for 24 and 48 h at 37°C ± 1°C. Before and after cultivation, the plates were mixed at 492× *g* for 10 min using a MixMate vortex shaker (Eppendorf, Hamburg, Germany). A mixture of alcohol and ether (1:1) was used to fix biofilm preparations on the glass surface for 10 min. Optical microscopy was performed by staining the preparations with an aqueous solution of Gentian Violet at a dilution of 1:2000 (HiMedia™). Preparations for optical and scanning electron microscopy were fixed with vapors of 25.0% glutaraldehyde solution for 8 h and then with vapors of 1.0% osmium tetroxide solution for 4 h [32].

Morphometric studies were performed using a representative sample with a reliable frequency of occurrence of ≥90.0% in the field of view of the Trinocular Unico optical microscope (Unico, New Jersey, USA). Furthermore, the samples were sputtered with gold ions Q150T ES (Quorum Technologies, London, Great Britain) for scanning electron microscopy analysis using Hitachi TM3030 Plus (Hitachi, Tokyo, Japan).

### Experimental design

White Leghorn chickens were maintained under the same conditions as those described in a previous study by Lenchenko *et al*. [32]. Experimental reproduction of the infectious process was performed on 10-day-old White Leghorn chickens (n = 20). Before the experiments, female birds were intranasally inoculated with 0.5 cm^3^ of 0.9% NaCl solution in Group I (control, n = 10) and intranasally inoculated with a suspension of *S. aureus* bacteria, 0.5 cm^3^, at a concentration of 1 billion/cm^3^.

Intravitally, 1.0 g of feces was collected from birds; postmortem samples were collected from hearts with ligated vessels, lungs, tubular bone, liver with gallbladder, and small intestine fragments. Inoculations were prepared from the pathological material on the middle surface of Petri dishes using a Pasteur pipette and evenly ground with a glass spatula. Microbiological cultures were also prepared using the “smear-print” method. The study organ samples were first flambéed on a burner flame and then cut with a sharp scalpel. A sterile spatula was used to evenly distribute the cut surface to the surface of the nutrient medium in the center of the Petri dish. The morphological, cultural, and biochemical properties of microbial strains isolated from avian pathological material were studied using differential media and API test systems [29, 30].

### Histopathological analysis

Samples for histopathological examination, including all layers of the organ, were collected. To study the stages of the pathological process development, samples were cut out in areas bordering tissue changes and those without changes. These samples were fixed in a 10.0% solution of neutral formalin at 22 ± 1°C for at least 48 h. We embedded the fixed material in paraffin and prepared histological sections on a microtome in accordance with standard histological procedures [11, 13, 18, 32]. Histological sections were stained with hematoxylin and eosin. Optical microscopy was used to measure the morphological parameters.

### Statistical analysis

The average values and standard deviations of the optical densities, adhesive properties of bacteria, and phagocytic activity of hemocytes were calculated using Microsoft Excel 2010 (Microsoft Office, Washington, USA). Student’s t-test was used to determine the difference between the mean samples and the control values, and the statistical significance of the differences was established at the level of p ≤ 0.05.

## Results

### Phenotypic characterization of bacteria

The morphological, tinctorial, and biochemical characteristics of the bacterial strains isolated from pathological material of experimentally infected birds corresponded to those of the reference strain. *S. aureus* cultures were Gram-positive, non-motile, and catalase- and coagulase-positive. Black S-form colonies with a diameter of 0.5–0.8 mm were formed on the surface of the Baird–Parker agar differential media for *Staphylococcus* spp. *S. aureus* fermented glucose and Mannitol and produced ammonia but did not ferment dulcite, salicin, or inulin.

### Morphometric and densitometric indicators of bacterial biofilm formation

Using optical and scanning electron microscopy methods, we identified general patterns of the formation of heterogeneous biofilm structures after 24–48 h of cultivation in liquid nutrient-both medium. We identified the following stages of biofilm formation: adhesion, fixation, microcolonization, growth, and dispersion. Optical microscopy revealed various sizes of dark brown bacteria in the preparations using vapor fixation of glutaraldehyde and osmium tetroxide. The bacterial aggregates or conglomerates formed due to binary fission were located in different directions. A diffuse layer was formed on the surface of the substrate due to the cells attached to the substrate and the intercellular matrix. Gradual colonization of individual areas of the substrate (in our case glass) was observed.

**Figure-1:**
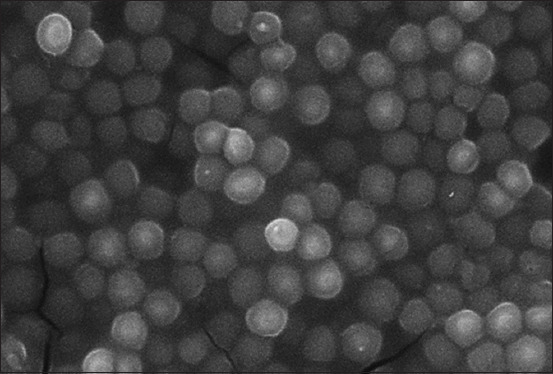
Culture of microorganisms *Staphylococcus aureus*, nutrient broth, 37 ± 1°C, 48 h: Round-shaped bacteria. Glutaraldehyde solution et Osmium tetraoxide solution. Scanning electron microscopy. Magnification: 2000× Hitachi TM3030 Plus (Japan).

Intercellular adhesion contributes to the attachment of other planktonic forms. We found pores and tubules between individual cell conglomerates, called clusters. Bacterial cells of different sizes, mostly coccoid in shape, were combined into short and long chains. Since we did not use liquid fixing solutions, it was possible to preserve the natural architecture of the biofilms when fixed with vapors of glutaraldehyde and osmium tetroxide (Figure-1). We observed individual sections of the substrate under SEM (Hitachi) to know how they were gradually populated.

### Clinical symptoms of *S. aureus* respiratory avian infection

Clinical signs of respiratory syndrome developed 5–6 days after infection. Depression, decreased appetite, sneezing, shortness of breath, discharge, and accumulation of liquid mucous exudate from the periorbital area and nasal openings were observed. Diarrhea and dehydration, along with respiratory syndrome, developed 7–8 days after infection.

### Confirmation of experimental microbiological infection findings

**Figure-2:**
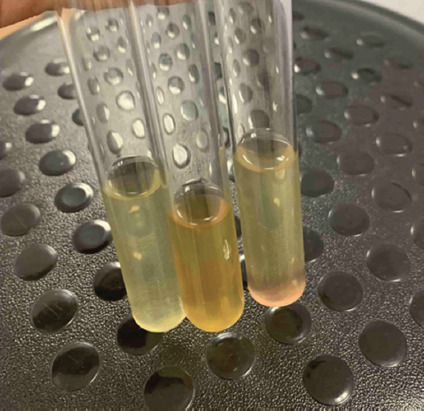
Cloudiness of nutrient broth due to growth *Staphylococcus aureus*, inoculating a drop of blood from an infected chicken, 37 ± 1°C, 24 h. A slight redness at the bottom of the tube is sediment from red blood cells.

**Figure-3:**
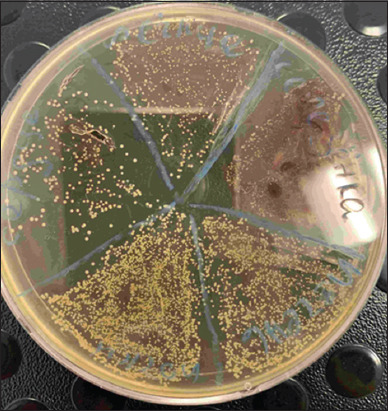
Growth of *Staphylococcus aureus* bacteria on a solid nutrient medium, 37 ± 1°C, 24 h. The petri dish is divided into five sectors. Each sector represents a culture from a sample of one internal organ (lungs, small intestine, liver, kidneys, or spleen) from one bird.

A pure culture of *S. aureus* was isolated from blood samples 5–10 days after infection (Figure-2) in the liquid nutrient broth. We isolated the same bacteria from the lungs, small intestine, liver, kidneys, and spleen on solid nutrient agar (Figure-3).

### Anatomopathological and histological changes of infected chickens

During the postmortem examination of the bird corpses, signs of cachexia and dehydration were observed. The subcutaneous and intermuscular connective tissues did not contain fat. Adipose tissue accumulation in the thoracoabdominal cavity or in the coronary sulcus of the heart and mesentery was not observed. Skin and soft tissues were swollen, reddened, and covered with dried exudate crust. The feather cover was sticky and wet in the periorbital area and around the nasal opening. Acute serous and serous-catarrhal rhinitis, sinusitis, laryngitis, and tracheitis were generally observed. Multiple small hemorrhages and swelling of the gelatinous consistency mucous membranes of the respiratory organs developed. The mucous membranes of the larynx and trachea were also swollen and reddened, and pinpoint hemorrhages were detected. Foamy exudate and semi-liquid mucous mass accumulation were detected in the tracheal cavity.

Necrosis, mucous dystrophy, and desquamation of single-layer prismatic ciliated epithelium of the tracheal mucosa were revealed. Loose fibrous connective tissue of the lamina propria and submucosal layer was edematous and pronounced lymphocyte and pseudoeosinophil infiltration was detected. Signs of inflammatory hyperemia development in blood vessels were identified.

Acute and subacute serous-fibrinous airsacculitis, characterized by edema and thickening of the membranes of the air sacs and the presence of turbid, watery, foamy contents in the cavity, was the most characteristic pathomorphological sign. Serous-fibrinous exudate was detected in the membranes of the air sacs in the form of a pale gray coating. Histological changes were accompanied by dystrophic processes and respiratory epithelial cell desquamation. The mucous membrane of the trachea was swollen, and fibrinous masses containing a significant number of pseudoeosinophils and erythrocytes were observed. In addition, we detected the proliferation of poorly differentiated connective tissue cells. The air sacs were multifocally dilated with lymphocytes, plasma cells, heterophilic macrophages, hemorrhages, and fibrin deposits. A mixed infiltrate was detected in the parenchyma of the air sac. Hyperemia, leukocyte infiltration, proliferation of fibroblasts, and perivascular loose fibrous unformed connective tissue were observed.

**Figure-4:**
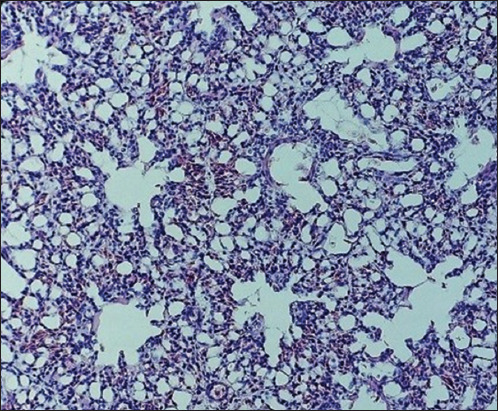
Lung of bird ♀, control: the contours of the bronchioles and alveoli are clearly defined, the blood vessels are moderately filled with blood. Hematoxylin and eosin, optical microscopy, 400× (“H604 Trinocular Unico”, USA).

**Figure-5:**
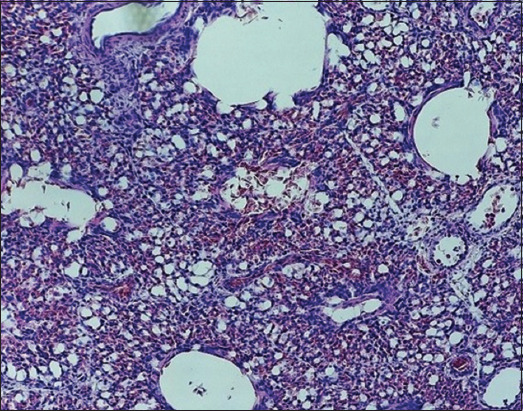
Lung of bird ♀ infected with *Staphylococcus aureus*. Experiment, 10 days of age: The lumens of the bronchioles and alveoli are filled with exudate, the blood vessels are filled with blood, and the interalveolar tissue is infiltrated with leukocytes. Hematoxylin and eosin, optical microscopy, 400× (“H604 Trinocular Unico”, USA).

**Figure-6:**
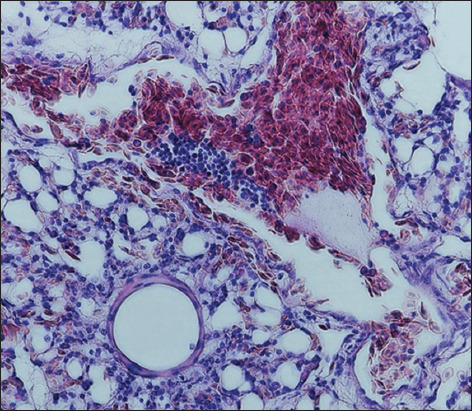
Lung of bird ♀ infected with *Staphylococcus aureus*. Experiment, 10 days of age: Blood vessels are stretched and filled with blood, and most red blood cells are stuck together. An accumulation of many leukocytes and pink exudate is detected. Hematoxylin and eosin, optical microscopy, 400× (“H604 Trinocular Unico”, USA).

Signs of fibrinous pleurisy were accompanied by the thickening of the opaque pleura. Gelatinous masses of gray–yellow color were observed under the pleura. The signs of acute congestive hyperemia and one-sided serous-fibrinous pneumonia developed with significant thickening of fibrinous deposits. The affected lungs were swollen and unevenly colored in red and blue shades. The hilar areas of the lungs were dark red and had a dense consistency. The areas of necrosis were surrounded by bright red rounded areas of demarcation inflammation. Histological changes were characterized by severe hyperemia of the pulmonary vessels, serous-fibrinous exudate-filled air capillary cavities and numerous pseudoeosinophils and adherent red blood cells. Multiple areas of coagulation necrosis were surrounded by areas of reactive inflammation. The lumens of the alveoli and bronchioles were partially filled with a pink film, and blood was filled with transudate, erythrocytes, single lymphocytes, pseudoeosinophilic desquamated alveolar epithelial cells, respiratory capillaries, and veins. Connective tissue edema and collagen fiber thickening near the vessels and bronchi were noted. Diffuse venous stasis, serous-inflammatory edema, and lymphocytic and pseudoeosinophilic infiltration of loose fibrous connective tissue were detected (Figures-4–6).

Signs of acute congestive hyperemia and the development of inflammatory processes are characterized by a significant accumulation of cloudy reddish fluid in the body cavity. Acute dilatation of the right cavity of the heart with stretching of the heart membranes and overflow of the pericardium with fibrinous-caseous masses developed. The myocardium is flabby and dark red. Serous-catarrhal gastroenteritis characterized by pronounced diffuse redness, swelling, and thickening of the membranes of the glandular and muscular stomach as well as the small and large intestines were observed. Pathological processes were accompanied by diffuse hemorrhagic inflammation of the cloacal mucosa. Signs of general venous stasis of the liver, hyperemia, diffuse focal protein-fatty dystrophy, and increased organ volume were observed. The pancreas was swollen, and small pinpoint hemorrhages were observed when the pancreas was cut. The volume of the kidneys has increased and the capsule has turned pale red. Splenic hyperplasia, which is characterized by a moderate increase in organ size and a fine-grained pulp surface on the cut, is a characteristic feature.

The cytopathological changes were generally similar to those observed with other bacterioses. In particular, dystrophic and necrotic processes developed in the thymus, Fabricius bursa, spleen, Harder’s glands, and Meckel’s lymphoid diverticulum. Karyorrhexis and lymphocyte karyopyknosis were detected in these organs of the immune system. Macrophage reactions were accompanied by pseudoeosinophilic leukocytes in the lymphoid follicle medulla. In Garder’s gland, there was an increase in the number of secretory sections, indicating hypersecretion of the glands. In the lymphoid follicles of Meckel’s diverticulum, leukocytes, usually lymphocytes, and pseudoeosinophils were detected.

## Discussion

The results of our studies showed that biofilm formation involves adhesion, fixation, microcolony formation, growth, and dispersion stages. The main stages of the *in vitro* development of biofilms of Gram-negative and Gram-positive bacteria and yeast-like fungi have been sufficiently studied and covered in detail in the literature [8, 33–35]. Effective methods for recording the bacterial mass and volume of the intercellular matrix of monospecies and polyspecies biofilms [32, 36] have been developed. After 1–2 h of interaction between *S. aureus* ATCC 29213 and human cell culture HT-29, most bacteria were located in the intercellular space. Colonization of the eukaryotic cell surface by bacterial cells was visualized after 3 h. Complete disorganization of the cell layer and destruction of eukaryotic cells was observed after 24 h [33]. Due to the growing resistance of *S. aureus* to various antibiotics, great efforts have been made to identify new antibacterial compounds. Although the mechanism by which biofilms are completely eradicated is not fully understood, researchers are seeking potential drug candidates as adjuvant therapeutics to treat or prevent biofilm-associated infections and to reverse resistance to existing antimicrobials. For example, lactic acid bacteria strains that produce bacteriocins and inhibit biofilms of MRSA strains are recommended to optimize therapy with antibacterial drugs to achieve maximum effectiveness and minimize side effects of resistance [37]. Mathematical models of understanding the relationship between the kinetics and dynamics of drug dosing regimens are considered promising to indicate control points of sensitivity and prevent resistance of microorganisms [38]. Laboratory models for studying the pathogenesis of infections and the dynamics of organ-specific immune responses provide a unique opportunity to develop therapeutics for the elimination of *Staphylococcal* pathogens [39]. Biofilm formation during experimental reproduction in living models has not been sufficiently investigated [32, 40]. Using a chick embryo model, the presence of numerous red blood cells with damaged cell membranes could be demonstrated; changes in heat stress protein 70 protein levels may be a useful indicator of staphylococcal infection [41].

The evolution of antibiotic resistance in *S. aureus* is associated with high genome variability, which can consist of phages, plasmids, transposons, and genomic islands. Due to the difficulty in detecting bacterial genome changes, the contribution of transformation or transduction to antimicrobial resistance gene transfer in clinical settings or in the environment requires further *in* *vivo* studies [42, 43]. The search for promising drugs is based on data on the functioning of quorum sensing (QS) systems of pathogenic micro-organisms [44–46], which are effector proteins from which protease toxins, adhesin proteins, proteins, and hemolysins are formed [47, 48].

Antibacterial drugs that will be developed to suppress the activity of the QS mechanisms of *S. aureus* bacteria will take their rightful place in anti-staphylococcal therapy. The testing and identification of standardized multi-level algorithms for the indication and differentiation of biofilms in the early stages of diagnosis will contribute to the development of preventive measures to control critical technology points and manage dangerous risk factors for infectious diseases. In addition to applied aspects, these studies will contribute to the expansion of theoretical knowledge about the general and specific pathology of birds with infectious bacterial diseases and in the long-term to the discovery of the mechanisms of adaptation of pathogenic microorganisms to long-term persistence in the form of biofilms *in vivo* and *in vitro*.

## Conclusion

Hydration and heteromorphism of the internal environment of biofilms determine the localization of differentiated cells in a three-dimensional matrix for protection from adverse factors. Pure cultures of *S. aureus* microorganisms were isolated after 5–10 days from the blood, lungs, small intestine, liver, kidneys, and spleen when reproducing the infectious process in susceptible models. Acute and subacute serous-fibrinous airsacculitis was the most characteristic pathomorphological sign. The signs of acute congestive hyperemia and one-sided serous-fibrinous pneumonia developed with significant thickening of fibrinous deposits.

## Authors’ Contributions

EL and NS: Conceptualization, methodology, investigation and writing–original draft preparation. OP and NP: Collected the samples. AZ: Data analysis and data cleaning. NZ and MA: Validation and writing–review and editing. All authors have read, reviewed, and approved the final manuscript.
